# HIF-1α inhibition by siRNA or chetomin in human malignant glioma cells: effects on hypoxic radioresistance and monitoring via CA9 expression

**DOI:** 10.1186/1471-2407-10-605

**Published:** 2010-11-04

**Authors:** Jacqueline Kessler, Antje Hahnel, Henri Wichmann, Swetlana Rot, Matthias Kappler, Matthias Bache, Dirk Vordermark

**Affiliations:** 1 Department of Radiotherapy, Faculty of Medicine, Martin-Luther-University Halle-Wittenberg, Ernst-Grube-Straße 40, 06097 Halle (Saale), Germany

## Abstract

**Background:**

Hypoxia induces activation of the HIF-1 pathway and is an essential characteristic of malignant gliomas. Hypoxia has been linked to tumor progression, therapy resistance and poor prognosis. However, little is known about the impact of HIF-1α inhibition on radioresistance of malignant glioma.

**Methods:**

In this study, we investigated the effects of the inhibition of HIF-1α on cell survival and radiosensitivity in U251MG and U343MG glioma cells, using two different strategies. HIF-1α inhibition was achieved by siRNA targeting of HIF-1α or via chetomin, a disruptor of interactions between HIF-1α and p300. The inhibition of the HIF-1 pathway was monitored by quantitative real-time PCR and Western blot analyses of the expression levels of HIF-1α and CA9. CA9 expression was investigated as a potential indicator of the efficacy of HIF-1 inhibition and the resulting radiosensitivity of malignant glioma cell lines was determined by clonogenic assay after irradiation under normoxic (2-10 Gy) or hypoxic (2-15 Gy) conditions.

**Results:**

Although siRNA and chetomin show distinct modes of action, both attenuated the hypoxia-induced radioresistance of malignant glioma cell lines U251MG (DMF_10_: 1.35 and 1.18) and U343MG (DMF_10_: 1.78 and 1.48). However, siRNA and chetomin showed diverse effects on radiosensitivity under normoxic conditions in U251MG (DMF_10_: 0.86 and 1.35) and U343MG (DMF_10_: 1.33 and 1.02) cells.

**Conclusions:**

Results from this *in vitro *study suggest that inhibition of HIF-1α is a promising strategy to sensitize human malignant gliomas to radiotherapy and that CA9 could serve as an indicator of effective HIF-1-related radiosensitization.

## Background

Malignant gliomas are tumors of the central nervous system originating from glial cells or their progenitors. According to the WHO classification, malignant gliomas are distributed in grade-III and grade-IV tumors [1,2]. Histologically characterized as pleomorphic, infiltrative tumors with microvascular proliferation and high mitotic rates, these cells show poor response to treatment [3]. Hence, patients with gliomas have a mean life expectancy of approximately one year in clinical trials, despite surgery, chemo- and radiotherapy [4]. With increasing malignancy, gliomas exhibit intratumoral hypoxia [5], which has been associated with poor responses to radio- or chemotherapy [6,7]. The transcription factor hypoxia inducible factor-1 (HIF-1), a dimer of HIF-1α and HIF-1β, is a critical mediator of the response to hypoxia. HIF-1 governs cellular adaption to oxygen deficiency by regulating tumor-relevant genes involved in energy metabolism, angiogenesis, cell proliferation and apoptosis [8-10]. Overexpression of HIF-1α promotes tumor growth, whereas the loss of HIF-1α activity dramatically decreases tumor growth, vascularization and energy metabolism [11]. Suppression of HIF-1α expression via antisense oligonucleotides was reported to reduce the survival of glioblastoma cells and accelerate p53-independent apoptosis [12]. In addition, knockdown of HIF-1α by RNA interference attenuates human glioma cell growth *in vivo *[13]. Furthermore, downregulation of HIF-1α by siRNA increased the sensitivity of human brain glioma cells to doxorubicin and etoposide [14].

HIF-1 activity can also be inhibited by chetomin (CTM), an epidithiodiketopiperazine metabolite of the fungal species *Chaetomium *[15]. Treatment with CTM attenuates hypoxia-inducible gene expression via reduction of the HIF-1α/p300 complex. At the molecular level, CTM disrupts the interactions of the C-terminal transactivation domain (TADC) of HIF-1α with the CH1 domain of p300, a transcriptional coactivator [15]. Previous studies have revealed that HIF-1α inhibition by CTM significantly reduced CA9 and VEGF mRNA expression and enhances radiation response under severely hypoxic conditions in human HT 1080 cells [16].

In the present study, we analyzed the inhibitory effects of two alternative HIF-1 targeting strategies, HIF-1α-siRNA and CTM, on HIF-1α expression and that of its target gene carbonic anhydrase 9 (CA9) in human malignant glioma cells. Further, we investigated whether targeting HIF-1α affects the hypoxia-induced radioresistance in these tumor cells.

## Methods

### Cell Culture Conditions and Treatments

Early-passage human glioma cell lines U251MG and U343MG (American Type Culture Collection) were grown in RPMI 1640 medium (Lonza, Walkersville, MD, USA) containing 10% fetal bovine serum, 1% sodium pyruvate, 185 U/ml penicillin and 185 μg/ml streptomycin at 37°C in a humidified atmosphere containing 3% CO_2_.

Gene silencing by small interfering RNA (siRNA) was carried out by transfection using HIF-1α-directed or control (Luciferase GL2) double-stranded RNA oligonucleotides. HIF-1α and Luciferase (Lu) siRNA were synthesized by Eurofins MWG Operon (Ebersberg, Germany). The target sequences are depicted in additional file 1: "siRNA Target Sequences". For siRNA experiments, cells (1.5 × 10^5^) were seeded in 12.5 cm^2 ^flasks 24 h before treatment with siRNA. At the time of transfection, the confluency of the monolayer was 40-50%. Different concentrations and (pre-) incubation times of siRNA were analyzed in pilot experiments. Transfection of 75 nM siRNA in U251MG and U343MG cells was carried out using Interferin (polyplus-transfection Inc., NY, USA) following the manufacturer's instructions. Cells were pre-treated with siRNA for 4 h at 37°C under normoxic conditions. Then cells were transferred for 20h to hypoxic conditions or maintained in a normoxic environment.

CTM (Alexis Biochemicals, Germany) was dissolved in 0.1% dimethyl sulfoxide (DMSO) for a 100 mM stock solution. Cells (3 × 10^5^) were seeded in 25 cm^2 ^flasks 24 h before treatment with 75 nM CTM. At the time of treatment, the confluency of the monolayer was 40-50%. Different concentrations and (pre-) incubation times of CTM were analyzed in pilot experiments. Cells were treated with CTM or DMSO for 4 h at 37°C under normoxic conditions. Then cells were transferred to hypoxic conditions or maintained in a normoxic environment and incubated for 20 h.

### Hypoxia and Irradiation

Hypoxia (<1% O_2_) was achieved using a gas generator system as previously described [17]. Four hours after siRNA transfection or incubation with CTM, the flasks with the treated or untreated cells were transferred into gas-proof plastic bags and cultured for 20 h under hypoxic conditions.

Irradiation was delivered by a linear accelerator using 6 MV photons with adequate bolus material at a dose rate of 200 MU/min. When normoxic conditions were applied, the cells treated with siRNA or CTM were irradiated with a single dose of 2-10 Gy at room temperature. Cells under hypoxia treated with siRNA or CTM were irradiated inside a plastic bag with a single dose of 2-15 Gy at room temperature. After irradiation, the cells were incubated under normoxia or hypoxia at 37°C for 1 h before harvest for the clonogenic assay, RNA isolation or protein isolation.

### Clonogenic Assay and Radiosensitivity

Clonogenic Assay was performed as previously described [17]. The surviving fractions were determined as ratios of the plating efficiencies (PE = counted colonies/seeded cells*100) of the irradiated cells to the non-irradiated cells. The dose-modifying factor at a 10% survival level (DMF_10_) was established to analyze the effects of siRNA or CTM on the radiosensitivity of glioma cells (DMF_10 _= radiation dose untreated cells/radiation dose treated cells). To fit the data a linear quadratic model lnS = -(α*D *+ β*D*^2^) was applied, using origin 7.5.

### Quantitative Real-Time PCR

Cells were washed twice with ice-cold phosphate buffered saline (PBS) and harvested using a cell scraper. RNA was isolated by using the RNeasy Mini Kit according to the manufacturer's instructions (QIAGEN, Hilden, Germany). Reverse transcription was carried out as described in additional file 2: "Reverse Transcription". Conditions for the quantitative real-time PCR (qRT-PCR) are depicted in additional file 3: "Conditions qRT-PCR". For normalization, hypoxanthine phosphoribosyltransferase (HPRT) was used as housekeeping gene. The primers for the amplification were synthesized by TIB MOLBIOL (Berlin, Germany), and their sequences are depicted in additional file 4:" Primer Sequences".

### Western Blot Analysis

Cells were washed twice with ice-cold phosphate buffered saline (PBS), harvested using a cell scraper and lysed with RIPA-buffer (50 mM Tris-HCl, 200 mM NaCl, 1 mM EDTA, 1 mM EGTA, 1% TritonX-100, 0.25% deoxycholate, protease and phosphatase inhibitors) 1 h after irradiation. The lysates were incubated on ice for 20 min and centrifuged for 10 min at 13,000 rpm at 4°C. The supernatants were collected and protein concentrations were determined using the Bradford method. Equivalent amounts of protein were resolved by electrophoresis in 4-12% Bis-Tris Mini Gels (Invitrogen) and then transferred to a PVDF membrane (Roth, Karlsruhe, Germany) by tank-electroblotting (Bio-Rad, München, Germany). Non-specific binding was blocked by Tris buffered saline (TBS) containing 10% nonfat powdered milk at room temperature for 1 h. The membrane was incubated with the primary antibody at the appropriate dilution in TBS-10% nonfat milk at 4°C overnight. The membrane was then washed three times with TBS and incubated with the appropriate horseradish peroxidise-conjugated secondary antibody at room temperature for 1 h. The protein-antibody complexes were detected by enhanced chemiluminescence (ECL-kit; Amersham, Freiburg, Germany). Details of the utilized antibodies are described in additional file 5:"Antibodies".

### Statistical Analyses and Figures

**Figure 1:**
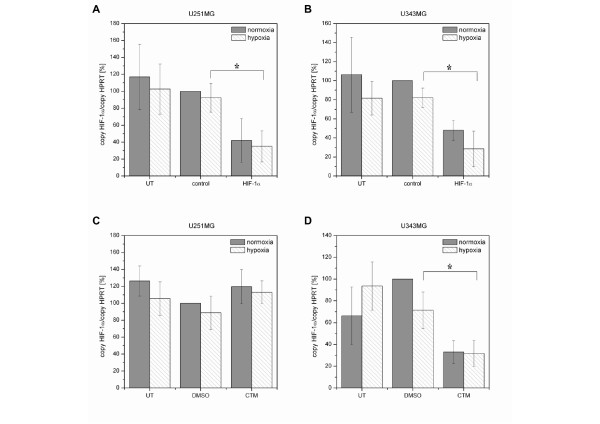
**Effects of HIF-1α-siRNA and CTM in U251MG and U343MG Cells: qRT-PCR of HIF-1α mRNA Levels**. **A (U251MG) and B (U343MG)**, Silencing of normoxic and hypoxic HIF-1α mRNA levels using 75 nM HIF-1α-specific siRNA in comparison with 75 nM control siRNA treated and untreated cells. **C (U251MG)**, CTM slightly enhanced the HIF-1α mRNA expression under normoxic and hypoxic conditions. 75 nM CTM in comparison with DMSO-treated and untreated cells. **D (U343MG)**, CTM decreased the normoxic and hypoxic HIF-1α mRNA expression. 75 nM CTM in comparison with DMSO-treated and untreated cells.

**Figure 2:**
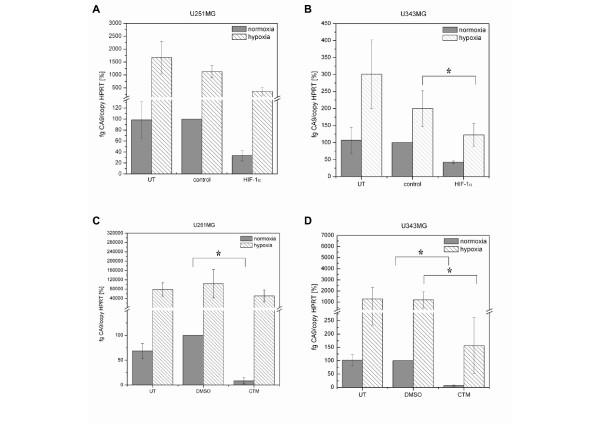
**Effects of HIF-1α-siRNA and CTM in U251MG and U343MG Cells: qRT-PCR of CA9 mRNA Levels**. **A (U251MG) and B (U343MG)**, Silencing of HIF-1α depleted the normoxic and hypoxic CA9 mRNA levels. CA9 mRNA levels using 75 nM HIF-1α-specific siRNA as compared to 75 nM control siRNA-treated and untreated cells. **C (U251MG) and D (U343MG)**, CTM depleted the normoxic and hypoxic expression of CA9 mRNA. 75 nM CTM in comparison with DMSO-treated and untreated cells.

Results are presented as means ± SD of n = 3 independent experiments. Statistical analyses were performed by Student's *t*-test. A *p*-value < 0.05 was considered significant. Results of figures (1;2;4) are expressed as a percentage of control (75 nM control siRNA/75 nM DMSO under normoxic conditions)

## Results

### Effects of HIF-1α-siRNA or CTM on HIF-1α and CA9 mRNA Expression in U251MG and U343MG Cells

In both U251MG and U343MG cells, neither hypoxia nor control siRNA or DMSO had an effect on HIF-1α mRNA expression. Twenty-four hours after treatment, the effect of the HIF-1α-siRNA or CTM on the HIF-1α mRNA levels was analyzed under normoxic and hypoxic conditions (Figure 1). Treatment of U251MG cells with the HIF-1α-siRNA resulted in an inhibition of HIF-1α mRNA by 58% (*p = *0.07, not significant) or 63% (*p *= 0.005) under normoxic or hypoxic conditions, respectively (Figure 1A). In U343MG cells, HIF-1α-siRNA decreased the HIF-1α mRNA levels by 52% (*p = *0.17, not significant) or 67% (*p *= 0.01) under normoxic or hypoxic conditions, respectively (Figure 1B).

Exposure to CTM had no effect on HIF-1α mRNA expression in U251MG cells (Figure 1C). However, treatment of U343MG cells with CTM resulted in 67% (*p *= 0.002) and 56% (*p *= 0.029) inhibition of HIF-1α mRNA under normoxic and hypoxic conditions (Figure 1D).

Inhibition of the HIF-1 activity was investigated by monitoring the expression of the HIF-1α target gene carbonic anhydrase 9 (Figure 2). In both U251MG and U343MG cells, hypoxia induced an increase in CA9 mRNA expression. Furthermore, neither control siRNA nor DMSO had an effect on the CA9 mRNA expression. Twenty-four hours after treatment, the effect of the siRNA or CTM on the CA9 mRNA levels was analyzed under normoxia and hypoxia (Figure 2). Effects of the HIF-1α-siRNA and CTM on HIF-1α and CA9 mRNA levels were compared to the respective control condition, i.e., cells treated with control siRNA or the solvent DMSO. Knockdown of HIF-1α by siRNA in U251MG cells caused a 67% (*p = *0.14, not significant) or 68% (*p *= 0.06, not significant) decrease in the CA9 mRNA expression under normoxic or hypoxic conditions (Figure 2A). A reduction in the CA9 mRNA expression by 57% (*p *= 0.12, not significant) or 38% (*p *= 0.01), accomplished by HIF-1α knockdown, was also observed under normoxia or hypoxia in U343MG cells (Figure 2B).

In U251MG cells, inhibition of HIF-1α activity in response to CTM caused a 92% (*p = *0.002) or 51% (*p = *0.18, not significant) decrease in CA9 mRNA expression under normoxic or hypoxic conditions, respectively (Figure 2C). In U343MG cells, treatment with CTM caused 94% (*p = *0.04) and 87% (*p = *0.03) decrease of the CA9 mRNA expression under normoxic or hypoxic conditions (Figure 2D).

### Effects of HIF-1α-siRNA or CTM on HIF-1α, CAIX and PARP Protein Expression in U251MG and U343MG Cells

**Figure 3:**
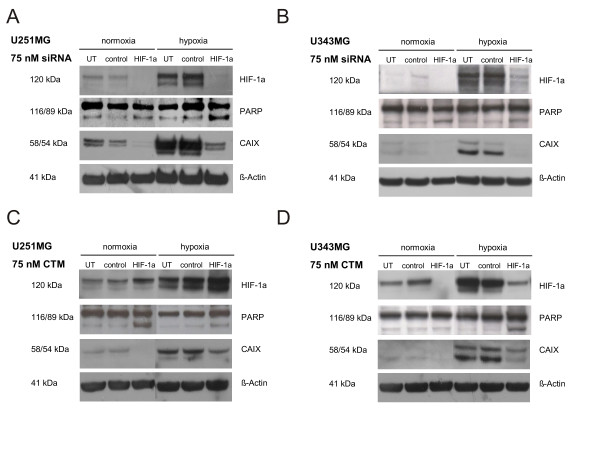
**Effects of HIF-1α-siRNA and CTM in U251MG and U343MG Cells: Western Blot Analyses HIF-1α, CAIX and PARP protein Levels**. HIF-1α/CAIX protein expression and PARP cleavage following treatment with 75 nM HIF-1α-siRNA/CTM or 75 nM control siRNA/DMSO in comparison with untreated cells. Specificity of siRNA on HIF-1α knockdown and effects of CTM on HIF-1 activity under normoxic and hypoxic conditions. **A (U251MG)**, Treatment with HIF-1α-siRNA caused induction of apoptosis under hypoxic conditions. CAIX expression is inhibited by HIF-1α silencing under normoxia and hypoxia. **B (U343MG)**, Transfection with HIF-1α-siRNA caused induction of apoptosis under normoxic and hypoxic conditions. CAIX expression is attenuated by HIF-1α silencing under normoxia and hypoxia. **C (U251MG)**, CTM enhanced the normoxic and hypoxic HIF-1α protein expression. Treatment with CTM induced apoptosis under normoxic and hypoxic conditions. CTM attenuated the normoxic and hypoxic CAIX expression. **D (U343MG)**, Treatment with CTM decreased the normoxic and hypoxic HIF-1α protein expression. Exposure to CTM caused induction of apoptosis under hypoxic conditions. CTM attenuated the normoxic and hypoxic expression of CAIX. β-Actin served as a loading control.

When we analyzed glioma cell lines, we found that, under normoxic conditions, U251MG and U343MG cells expressed small amounts of HIF-1α protein (Figure 3). Under hypoxia, HIF-1α protein levels were increased in both U251MG and U343MG cells. Treatment with control siRNA or DMSO did not affect HIF-1α protein expression (Figure 3). Treatment of U251MG and U343MG cells with the HIF-1α-siRNA strongly down-regulated HIF-1α protein expression under normoxic and hypoxic conditions (Figure 3A and 3B).

Exposure to CTM resulted in an increase of HIF-1α protein expression under normoxic and hypoxic conditions in U251MG cells (Figure 3C). In contrast to U251MG cells, the U343MG cells showed decreased levels of HIF-1α protein in response to CTM (Figure 3D).

The CAIX protein is expressed in both U251MG and U343MG cells under normoxic conditions. In response to hypoxia, CAIX protein levels were substantially increased in both cell lines (Figure 3). HIF-1α knockdown strongly decreased CAIX protein expression in both U251MG and U343MG cells, irrespective of whether cells were grown under normoxic or hypoxic conditions (Figure 3A and 3B).

In U251MG cells, inhibition of the HIF-1α transcriptional activity by CTM down-regulated the normoxic and hypoxic CAIX protein expression (Figure 3C). Furthermore, CTM reduced the normoxic and hypoxic CAIX protein expression in U343MG cells (Figure 3D).

The proteolytic cleavage of PARP via Western blot analysis qualitatively assess induction of apoptosis. HIF-1α knockdown using siRNA did not affect the normoxic expression of the large fragment of PARP, but did cause PARP cleavage under hypoxic conditions in U251MG cells (Figure 3A). In U343MG cells, HIF-1α knockdown induced apoptosis under normoxic and hypoxic conditions (Figure 3B).

Exposure to CTM caused induction of apoptosis under normoxic and hypoxic conditions in U251MG cells (Figure 3C). Furthermore, treatment with CTM had no effect on apoptosis under normoxic conditions but caused induction of apoptosis under hypoxic conditions in U343MG cells (Figure 3D).

### Effects of HIF-1α-siRNA or CTM on Clonogenic Survival and Radiosensitivity

**Figure 4:**
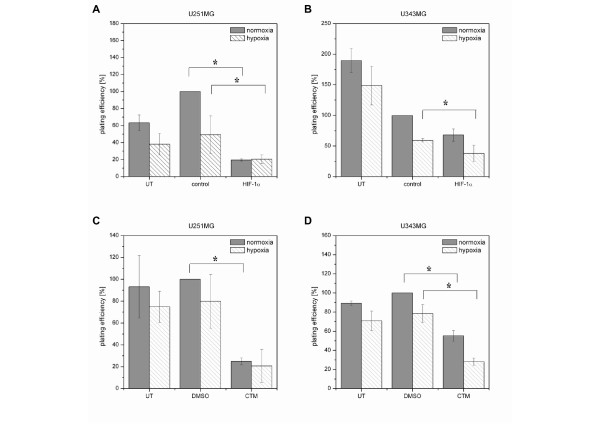
**Effects of HIF-1α-siRNA or CTM on Clonogenic Survival in U251MG and U343MG Cells (Cytotoxicity)**. **A (U251MG) and B (U343MG)**, Effects of treatment with the indicated siRNA (75 nM) on the plating efficiency of non-irradiated cells. **C (U251MG) and D (U343MG)**, Effects of treatment with 75 nM CTM on the plating efficiency of non-irradiated cells.

**Figure 5:**
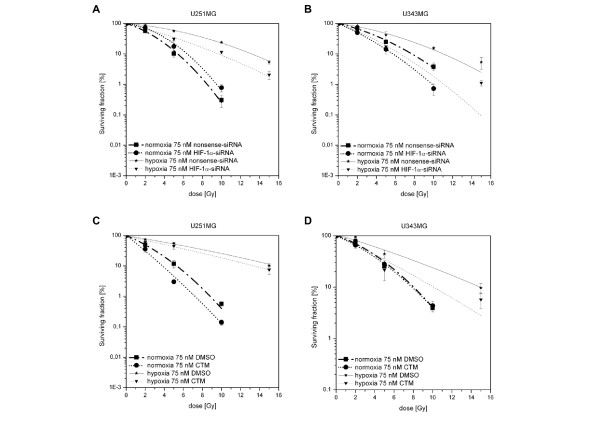
**Effects of HIF-1α-siRNA or CTM on Response to Radiation of U251MG and U343MG Cells (Radiosensitivity)**. **A (U251MG) and B (U343MG)**, Clonogenic survival following treatment with the indicated siRNA (75 nM) and radiation under normoxia (0-10 Gy) and hypoxia (0-15 Gy). HIF-1α-siRNA decreased the hypoxia-induced radioresistance of U251MG and U343MG cells. Under normoxia, HIF-1α-siRNA reduces the response to irradiation in U251MG cells and enhanced the effect of radiation in U343MG cells. **C (U251MG) and D (U343MG)**, Clonogenic survival following exposure to 75 nM CTM and radiation under normoxia (0-10 Gy) and hypoxia (0-15 Gy). Exposure to CTM decreased hypoxia-induced radioresistance of U251MG and U343MG cells. CTM enhanced the effect of radiation in U251MG cells and reduced the response to irradiation in U343MG cells under normoxia.

The clonogenic survival assay is a crucial endpoint read-out that we used to determine the fraction of cells surviving the treatment with the HIF-1α-siRNA or CTM (Figure 4) and the combined treatment of HIF-1α-siRNA or CTM and radiation (Figure 5).

Treatment with control siRNA or DMSO did not affect the plating efficiency of U251MG cells as compared to that of untreated cells (Figure 4A and 4C). In U343MG cells, a toxic effect of control siRNA was observed when compared to the untreated cells (reduction of PE by 40% under normoxia and by 60% under hypoxia), whereas DMSO did not affect the plating efficiency of U343MG cells (Figure 4B and 4D).

Treatment of U251MG cells with HIF-1α-siRNA reduced the plating efficiency under normoxic (80%) (*p = *0.04) and hypoxic (59%) (*p *= 0.04) conditions as compared to that of the control siRNA-treated cells (Figure 4A). Furthermore, treatment of U343MG cells with HIF-1α-siRNA decreased the plating efficiency under normoxic (32%) (*p *= 0.10, not significant) and hypoxic (36%) (*p = *0.05) conditions as compared to that of the control siRNA-treated cells (Figure 4B).

Treatment of U251MG cells with CTM reduced the plating efficiencies under normoxic (76%) (*p *= 0.02) and hypoxic (75%) (*p *= 0.07, not significant) conditions as compared to those the DMSO-treated cells (Figure 4C). Furthermore, treatment of U343MG cells with CTM decreased the plating efficiency under normoxic (45%) (*p *= 0.0008) and hypoxic (65%) (*p *= 0.004) conditions as compared to that of the DMSO-treated cells (Figure 4D).

The clonogenic assay was also applied to determine whether HIF-1α-siRNA or CTM sensitized glioma cells U251MG and U343MG to radiation. The viability of non-irradiated cells was set as 100%. Survival of U251MG and U343MG cells increased under hypoxic conditions as compared to normoxia (Figure 5). Survival curves revealed that, although HIF-1α-siRNA only slightly diminished the cytotoxic effect of radiation on U251MG cells under normoxic (DMF_10_: 0.86, *p *= 0.04) conditions, it significantly enhanced radiation sensitivity of U251MG cells under hypoxic conditions (DMF_10_: 1.35, *p = *0.002) (Figure 5A). Further, treatment with HIF-1α-siRNA resulted in a significantly enhanced radiosensitivity of U343MG cells under normoxic (DMF_10_: 1.33, *p = *0.002) and hypoxic (DMF_10_: 1.78, *p = *0.001) conditions (Figure 5B), respectively.

The survival curves indicate that exposure to CTM significantly enhanced the effect of radiation in U251MG cells under normoxic (DMF_10_: 1.35, *p *= 0.001) and hypoxic (DMF_10_: 1.18, *p *= 0.04) conditions (Figure 5C). Treatment with CTM did not affect radiosensitivity of U343MG cells under normoxic (DMF_10_: 1.02, *p *= 0.01) conditions, but resulted in significant enhanced radiosensitivity under hypoxic (DMF_10_: 1.48, *p *= 0.02) conditions (Figure 5D).

## Discussion

In most human cancers including malignant glioma, HIF-1α expression promotes tumor growth, angiogenesis and disease progression [18-20]. Additionally, the malignancy of brain tumors is associated with increased intratumoral hypoxia [5].

In the present study, we analyzed the effects of HIF-1α inhibition by two alternative strategies, siRNA targeting of HIF-1α or CTM, on the radioresistance of two malignant glioma cell lines. The glioma cell lines U251MG and U343MG displayed comparable HIF-1α mRNA expression levels under normoxic and hypoxic conditions. U251MG and U343MG exhibited a weak HIF-1α protein expression under normoxia with an increase in HIF-1α protein, CA9 mRNA and CAIX protein levels under hypoxia. Our previous studies using the glioma cell line U87MG supported the potential usefulness of HIF-1α as an endogenous marker of tumor hypoxia and of resulting radioresistance of human tumor cells [21,22]. Furthermore, CAIX was found to be a suitable marker of current or previous chronic hypoxia and we previously proposed that the amount of CAIX-positive cells in a tumor cell suspension may be related to the radiosensitivity of this tumor *in vivo *[23]. Inhibition of the HIF-1 pathway by siRNA or CTM was performed by quantitative real-time PCR and Western blot analyses. We showed that HIF-1 targeting via HIF-1α-siRNA effectively reduced the hypoxia-induced HIF-1α protein levels of U251MG and U343MG cells. In addition, exposure to CTM resulted in an increased HIF-1α protein expression in U251MG cells and decreased levels of HIF-1α protein in U343MG cells under normoxic and hypoxic conditions. CTM is an inhibitor of the HIF-1α/p300 complex. CTM only interrupts the transcriptional activity of HIF-1α. Thus, it diminishes the expression of HIF-1α target genes. U251MG cells responded as expected because CTM has no influence on the expression of HIF-1α mRNA. In U251MG cells, treatment with CTM resulted in a slight increase of HIF-1α protein stabilisation but attenuated the HIF-1α induced expression of CAIX. One possible explanation for the slightly increased HIF-1α protein expression in these cells might be that CTM attenuated the hypoxic answer. So an auto regulatory feedback loop caused this stabilisation of HIF-1α. Moreover, another explanation could be that CTM also inhibits the interaction of HIF-1α and VHL. In this, way an unspecific side effect of CMT would inhibit the degradation of HIF-1α.

U343MG cell line didn't respond as expected. In U343MG cells CTM has an effect on the HIF-1α expression and on the target gene CAIX. One reason for that might have been that the applied dose of CTM in the lower grade 3 glioma cells line U343MG causes side effects. Further, both cell lines differ in their genetic status and regulation of different pathways and consequently in their disease aggressiveness and response to treatment. Additionally, CTM is a small molecule and the specificity for the target of small molecule is debatable and there might be another mechanism of CTM play a role. Furthermore, our data indicate that monitoring CA9 mRNA and CAIX protein expression is a means to verify the inhibition of HIF-1 activity.

Results suggest that inhibition of HIF-1α decreased tumor growth in malignant gliomas [24]. Additionally, Dai *et al. *showed oxygen-independent cytotoxicity and p53-independent apoptosis by HIF-1α inhibition in glioblastoma cells [12]. In the present study, Western blot analyses of the cleavage of PARP confirm these findings. Furthermore, treatment of U251MG and U343MG cells with HIF-1α-siRNA or CTM reduced the plating efficiency under normoxic and hypoxic conditions as compared to that of the control siRNA- or DMSO-treated cells.

In the literature, hypoxia and HIF-1α-associated resistance to radiotherapy and chemotherapy is discussed. HIF-1α expression is a significant prognostic indicator in glial brain tumors treated by radiotherapy [18,25]. Furthermore, HIF-1α exhibits an attractive target to overcome hypoxia-related radioresistance of tumors *in vitro *and *in vivo *[26]. Silencing of HIF-1α has been shown to inhibit hypoxia-mediated cell migration and invasion in *in vitro *and *in vivo *malignant glioma models [13,27,28]. Further, silencing of HIF-1α by siRNA sensitized glioma cells to the chemotherapeutic agents doxorubicin and etoposide [14]. This is the first study showing that inhibition of HIF-1α by siRNA or by CTM under hypoxic conditions resulted in a significantly enhanced radiosensitivity of the human glioma cells. Recently, HIF-1α inhibition by CTM effectively reduced hypoxia-dependent transcription and sensitized hypoxic HT 1080 human fibrosarcoma cells *in vitro *to radiation [16].

Although inhibition of HIF-1α under hypoxic conditions enhanced radiosensitivity of glioma cells U251MG and U343MG, the effects of HIF-1α inhibition on radiosensitivity under normoxic conditions were not obvious. In agreement with our results, Palayoor *et al*. showed that effects of HIF-1α inhibitor PX-478 on radiosensitity under normoxic conditions were less dramatic than under hypoxic conditions in prostate carcinoma cells [29]. Further studies have shown that HIF inhibitors are more potent in attenuation of HIF-dependent transcriptional activity under hypoxia than under normoxia [30,31]. Further investigations are necessary to study the normoxic expression of HIF-1α in malignant glioma cell lines.

Indeed, siRNA and CTM showed different impacts on the effects of radiation in the indicated cell lines under normoxia, as HIF-1α-siRNA reduces the response to irradiation in U251MG cells, whereas in U343MG cells, siRNA enhanced the effects of radiation. Furthermore, under normoxia, CTM enhanced the effects of radiation in U251MG cells, whereas in U343MG cells, CTM had no effect on the response to irradiation under normoxia.

The U343MG cell line originated from a *de novo *grade-III anaplastic astrocytoma and U251MG was established from a *de novo *grade-IV glioblastoma [1,2,32]. Thus, different genes involved in cellular pathways regulating apoptosis, proliferation, and DNA repair could carry mutations or deletions in these cells. Both cell lines differ in their genetic status of p53 and consequently in their disease aggressiveness and response to treatment. U343MG cells carry wild-type p53, whereas U251MG carry mutated p53 [32]. Hence, the ability to respond to radiation may differ in U251MG and U343MG cells. Additionally, compared to HIF-1α-specific siRNA, CTM has a different mode of action.

However, the function of CTM is not fully understood. CTM is a member of the epidithiodiketopiperazine (ETP) family. Previous studies have shown that ETPs react with p300 and cause zinc ion ejection. Additionally, the results of Cook *et al*. suggest that ETPs also interact with other zinc ion binding proteins. In addition, it is likely that ETPs like CTM operate by more than one mechanism in cells and that CTM had an impact on transcription factors relevant to radiosensitivity [33].

## Conclusions

We demonstrate for the first time that both siRNA and CTM treatment decreased the radioresistance of glioma cell lines U251MG and U343MG under hypoxic conditions. Further, treatment of glioma cells lines with siRNA and CTM reduced plating efficiency of these cells and caused induction of apoptosis under hypoxic conditions. In conclusion, our results suggest that inhibition of HIF-1α is a promising strategy to reduce cell survival and to enhance the response of malignant gliomas to radiotherapy.

## Abbreviations

UT: untreated cells; control: control siRNA (luciferase) treated cells; HIF-1α: HIF-1α-siRNA treated cells; DMSO: DMSO treated cells; CTM: CTM treated cells

## Competing interests

The authors declare that they have no competing interests.

## Authors' contributions

JK designed the study, performed experimental procedures, analyzed the data and drafted the manuscript. AH HW SR and MK aided in study design and reviewed the manuscript. MB analyzed the data and reviewed the manuscript. DV designed the study, performed experimental procedures, analyzed the data and drafted the manuscript. All authors read and approved the final manuscript.

## Pre-publication history

The pre-publication history for this paper can be accessed here:

http://www.biomedcentral.com/1471-2407/10/605/prepub

## Supplementary Material

Additional file 1 **siRNA Target Sequences**. The file contains the target sequences of the utilized siRNA. Click here for file

Additional file 2 **Reverse Transcription**. The file contains the conditions for the reverse transcription. Click here for file

Additional file 3 **Conditions qRT-PCR**. The file contains the conditions for the qRT-PCR. Click here for file

Additional file 4 **Primer Sequences**. The file contains the sequences of the applied primer. Click here for file

Additional file 5 **Antibodies**. The file contains the details of the utilized antibodies. Click here for file
