# Supplementary material for: HIF-1α inhibition by siRNA or chetomin in human malignant glioma cells: effects on hypoxic radioresistance and monitoring via CA9 expression
Source: BMC Cancer. 2010 Nov 4;10:605. doi: 10.1186/1471-2407-10-605 (PMC2992520; doi:10.1186/1471-2407-10-605)
Supplement: Additional file 1 — siRNA Target Sequences. The file contains the target sequences of the utilized siRNA. [file 1471-2407-10-605-S1.PDF]

Additional file 1: siRNA Target Sequences

**Target mRNA**	**siRNA**	**Sequence 5'→3'**
HIF-1α	HIF-1α	5'-CUGAUGACCAGCAACUUGA-3' (sense)<br>5'-UCAAGUUGCUGGUCAUCAG-3' (antisense)
Luciferase	Lu GL2<br>control siRNA	5'-CGUACGCGGAAUACUUCGA-3' (sense)<br>5'-UCGAAGUAUUCCGCGUACG-3' (antisense)
