# Supplementary material for: HIF-1α inhibition by siRNA or chetomin in human malignant glioma cells: effects on hypoxic radioresistance and monitoring via CA9 expression
Source: BMC Cancer. 2010 Nov 4;10:605. doi: 10.1186/1471-2407-10-605 (PMC2992520; doi:10.1186/1471-2407-10-605)
Supplement: Additional file 2 — Reverse Transcription. The file contains the conditions for the reverse transcription. [file 1471-2407-10-605-S2.PDF]

Additional file 2: Reverse Transcription

**cDNA-Synthesis Mix**	
Superscript™ II RNaseH reverse transcriptase	200 U
dNTP mix	1 mM
buffer	4 µl
dithiothreitol	10 mM
random hexameric primers	1 µg
template	1 µg RNA
**Reverse Transcription**	
70°C	10 min
20°C	10 min
42°C	80 min
95°	10 min
