# Supplementary material for: HIF-1α inhibition by siRNA or chetomin in human malignant glioma cells: effects on hypoxic radioresistance and monitoring via CA9 expression
Source: BMC Cancer. 2010 Nov 4;10:605. doi: 10.1186/1471-2407-10-605 (PMC2992520; doi:10.1186/1471-2407-10-605)
Supplement: Additional file 3 — Conditions qRT-PCR. The file contains the conditions for the qRT-PCR. [file 1471-2407-10-605-S3.PDF]

Additional file 3: Conditions qRT-PCR

**qRT-PCR Mix**		
cDNA	1 µl	
SyBrGreen Quantitect	9 µl	
water	4.6 µl	
forward primer (20 µM)	0.2 µl	
reverse primer (20 µM)	0.2 µl	
**qRT-PCR**		
initial denaturation	at 95°C for 15 min	
denaturation	at 95°C for 30 sec	35 cycles
annealing	at 60°C for 30 sec	
extension	at 72°C for 30 sec	
