# Supplementary material for: HIF-1α inhibition by siRNA or chetomin in human malignant glioma cells: effects on hypoxic radioresistance and monitoring via CA9 expression
Source: BMC Cancer. 2010 Nov 4;10:605. doi: 10.1186/1471-2407-10-605 (PMC2992520; doi:10.1186/1471-2407-10-605)
Supplement: Additional file 4 — Primer Sequences. The file contains the sequences of the applied primer. [file 1471-2407-10-605-S4.PDF]

Additional file 4: Primer Sequences

Gene	Primer	Sequence 5’→3’
HPRT	HPRT 309<br>HPRT 570	5’-TTGCTGACCTGCTGGATTAC-3’<br>5’-CTTGCGACCTTGACCATCTT-3’
CA9	CA9 fw<br>CA9 bw	5’-GAAAACAGTGCCTATGAGCAGTTG-3’<br>5’-TGCTTAAGCACTCAGCATCAC-3’
HIF-1α	HIF-1α fw<br>HIF-1α bw	5’-CCACAGGACAGTACAGGATG-3’<br>5’-TCAAGTCGTGCTGAATAATACC-3’
