# Supplementary material for: HIF-1α inhibition by siRNA or chetomin in human malignant glioma cells: effects on hypoxic radioresistance and monitoring via CA9 expression
Source: BMC Cancer. 2010 Nov 4;10:605. doi: 10.1186/1471-2407-10-605 (PMC2992520; doi:10.1186/1471-2407-10-605)
Supplement: Additional file 5 — Antibodies. The file contains the details of the utilized antibodies. [file 1471-2407-10-605-S5.PDF]

Additional file 5: Antibodies

Antibody	Source	Dilution	Company
anti-HIF-1α	monoclonal mouse	1:1000	BD Transduction Laboratories, Lexington, KY
MN75 anti CAIX	monoclonal mouse	1:2000	Bayer Healthcare Co., Germany
anti-β-actin	monoclonal mouse	1:5000	Sigma, Steinheim, Germany
anti-PARP	monoclonal rabbit	1:1000	Cell Signaling, Germany
anti-mouse immunoglobulin G (IgG)-horseradish peroxidase conjugate	polyclonal rabbit	1:2000	DAKO, Hamburg, Germany
anti-rabbit immunoglobulin G (IgG)-horseradish peroxidase conjugate	polyclonal goat	1:2000	DAKO, Hamburg, Germany
